# Supplementary material for: Overexpression of NRF1-742 or NRF1-772 Reduces Arsenic-Induced Cytotoxicity and Apoptosis in Human HaCaT Keratinocytes
Source: Int J Mol Sci. 2020 Mar 16;21(6):2014. doi: 10.3390/ijms21062014 (PMC7139366; doi:10.3390/ijms21062014)
Supplement: Supplementary file 1 [file ijms-21-02014-s001.pdf]

Table S1. List of primers for qRT–PCR

Gene		Primer sequence(5'-3')	GenBank ID
β-ACTIN	Forward	GTCCACCTTCCAGCAGATGTG	NM_001101.5
	Reverse	GCATTTGCGGTGGACGAT	
NFE2L2	Forward	AACCAGTGGATCTGCCAACTACTC	NM_006164.5
	Reverse	CTGCGCCAAAAGCTGCAT	
KEAP1	Forward	CCTCTGGCCGGGTAATAGG	NM_012289.4
	Reverse	CCCCTCCCAGGTATCCAAGA	
NQO1	Forward	ACTGCCCTCTTGTGGTGCAT	NM_000903.3
	Reverse	GCTCGGTCCAATCCCTTCAT	
GCLC	Forward	GATGCTGTCTTGCAGGGAATG	NM_001498.4
	Reverse	AGCGAGCTCCGTGCTGTT	
GCLM	Forward	ACAGGTAAAACCAAATAGTAACAAAGTTAA	NM_002061.4
	Reverse	TGTTTAGCAAATGCAGTCAAATCTG	
PSMC3	Forward	CGAGCAAGATGGAATTGGGGA	NM_002804.4
	Reverse	GCTCATGGGTGACTCTCAACA	
PSMC4	Forward	GGACATCGGAGGCATGGAC	NM_006503.4
	Reverse	GGTGGGCCATACATGAGGAC	
XPC	Forward	CATCGTGGGAGCCATCGTAAG	NM_004628.4
	Reverse	CTCACCATCGCTGCACATTTT	

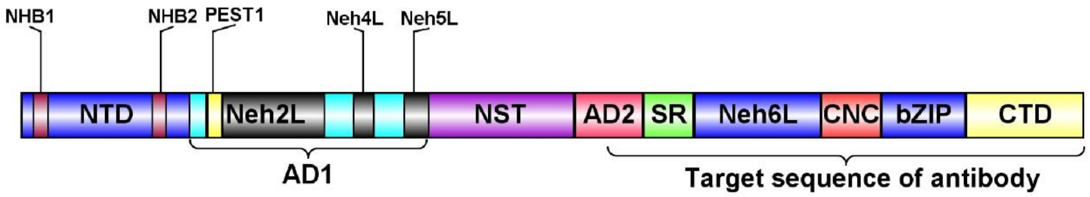

Figure S1. Schematic diagram of the human NRF1 protein structural domains. The N-terminal domain (NTD) contains the N-terminal homology box 1 and 2 (NHB1 and NHB2). Acidic Domain 1 (AD1) consists of Pro/Glu/Ser/Thr-rich (PEST1), Neh2-like (Neh2L), Neh4-like (Neh4L) and Neh5-like (Neh5L) domains. Asn/Ser/Thr-rich (NST), serine-repeat (SR), Neh6-like (Neh6L), Cap'N' Collar (CNC), Basic-leucine zipper (bZIP) and C-terminal domain (CTD) are behind in sequence. Target sequence of antibody is marked, which refers to 423-772 aa.

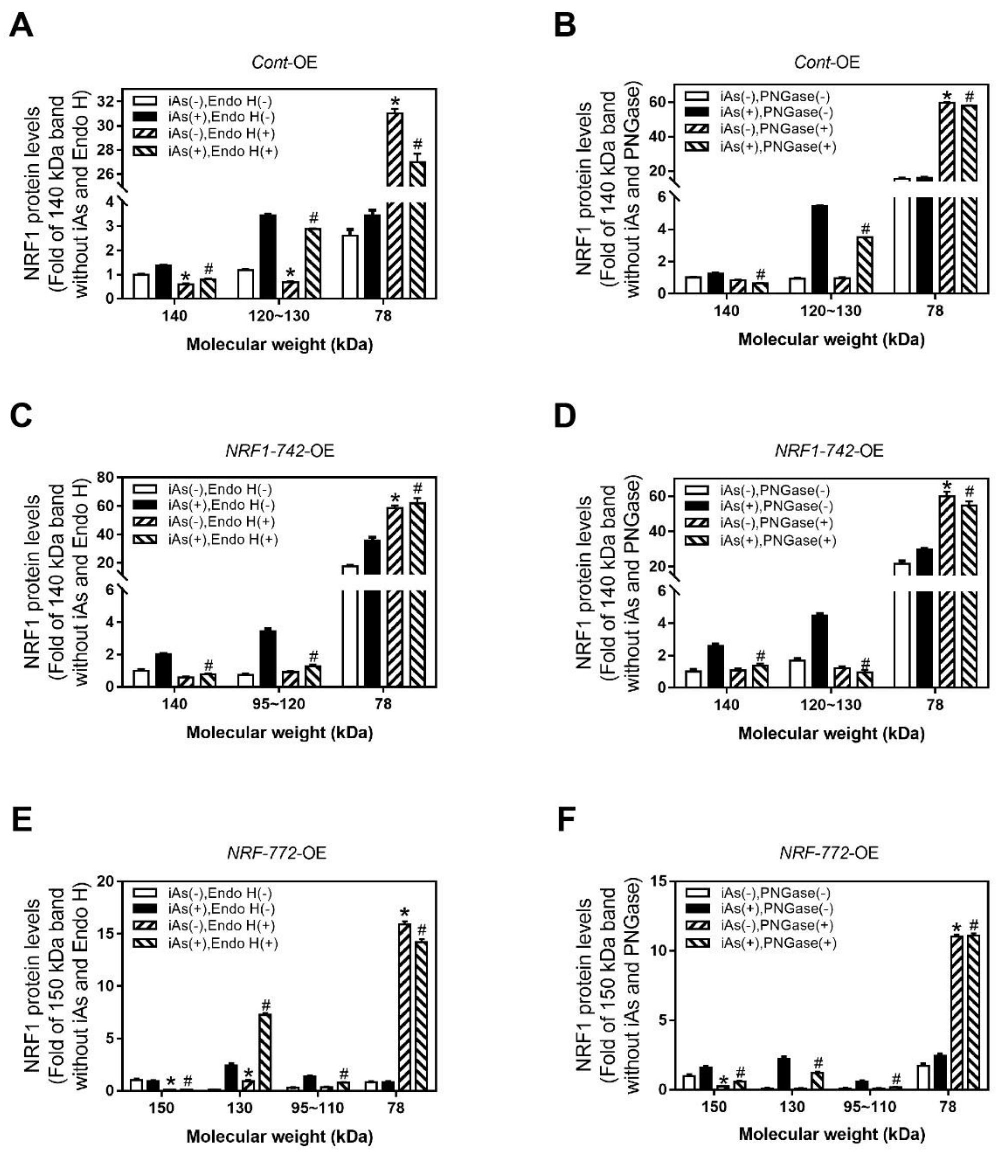

Figure S2. The grey density analysis of NRF1 bands with the treatment of Endo H or PNGase under iAs^{3+} treatment. (A-B) Quantification of NRF1 bands in *Cont*-OE cells with Endo H or PNGase respectively. (C-D) Quantification of NRF1 bands in *NRF1-742*-OE cells with Endo H or PNGase respectively. (E-F) Quantification of NRF1 bands in *NRF1-772*-OE with Endo H or PNGase respectively. The data are presented as the mean ± SD; $^{*}p < 0.05$, under normal conditions, bands with deglycosylase versus bands without deglycosylase; $^{\#}p < 0.05$, under iAs^{3+} treatment, bands with deglycosylase versus bands without deglycosylase.
